# MiR-195-5p Promotes Cardiomyocyte Hypertrophy by Targeting MFN2 and FBXW7

**DOI:** 10.1155/2019/1580982

**Published:** 2019-06-25

**Authors:** Lei Wang, Dongze Qin, Hongtao Shi, Yanan Zhang, Hao Li, Qinghua Han

**Affiliations:** ^1^Shanxi Medical University, Taiyuan 030001, Shanxi, China; ^2^The Affiliated Cardiovascular Hospital of Shanxi Medical University, Taiyuan 030001, Shanxi, China; ^3^Department of Cardiology, The First Hospital of Shanxi Medical University, Taiyuan 030001, Shanxi, China

## Abstract

Cardiac hypertrophy mainly predicts heart failure and is highly linked with sudden loss of lives. MicroRNAs (miRNAs) play essential roles in the development of cardiac hypertrophy through binding to corresponding mRNA targets. In this study, in order to investigate the roles of two mature forms of miRNA-195, miR-195-3p, and miR-195-5p,* in vitro* and* in vivo* models of cardiac hypertrophy were established by applying angiotensin II (Ang II) to H9c2 cardiomyocytes and infusing chronic Ang II to mice, respectively. We found that miR-195-5p was evidently equally upregulated in the* in vitro *and* in vivo* studies of cardiac hypertrophy induced by Ang II. High expressed miR-195-5p could adequately promote hypertrophy, whereas the suppression of miR-195-5p prevented hypertrophy of H9c2 cardiomyocytes under Ang II treatment. Furthermore, the luciferase reporter system demonstrated that MFN2 and FBWX7 were target genes of miR-195-5p, which negatively regulated the expression of these two genes in H9c2 cells. By contrast, in both models, expression of miR-195-3p was only slightly changed without statistical significance. In addition, we observed a trend towards decreased expression of hypertrophic markers by overexpressing miR-195-3p in AngII-treated H9c2 cardiomyocytes* in vitro*. Taken together, our study indicates that miR-195-5p promotes cardiac hypertrophy via targeting MFN2 and FBXW7 and may provide promising therapeutic strategies for interfering cardiac hypertrophy.

## 1. Introduction

As a common adaptive reaction towards diverse physiologic and pathologic derived stimulus, cardiac hypertrophy involves mechanical, hormonal, and hemodynamic factors. The major cellular basis of cardiac hypertrophy includes increased cardiomyocyte size, actin cytoskeletal reorganization, and re-expression of fetal genes [1]. As cardiac hypertrophy is the most important determinant of heart failure and highly associated with sudden cardiac death [2], a greater understanding of the underlying molecular mechanism, especially the roles of hypertrophy mediators, may reveal novel therapeutic targets and thereby improve survival.

MicroRNAs (miRNAs) are noncoding single-stranded RNAs made up of 17–25 nucleotides that can control expression of a gene when they bind mRNAs at the 3′ untranslated region (UTR), inhibiting protein synthesis [3]. In recent years, increasing evidence suggests significant roles played by miRNAs in cardiovascular diseases. For example, miR-124 [4], miR-21 [5], and miR-497 [6] have been reported to be critically involved in myocardial infarction, fibrosis, and cardiac hypertrophy. As such, accumulating experimental data [7] have demonstrated that miRNAs have therapeutic potential in the treatment of cardiac system disorders, especially cardiac hypertrophy.

It is well known that precursor miRNAs can be cleaved from both the 5′ and 3′ arms of the precursor duplex, becoming the miRNA-5p and -3p strands, respectively [8]. Previously, one of the strands was thought to be a biologically functional miRNA, and the other was considered as an inactive passenger strand [9]. Nevertheless, recently, many passenger miRNAs have identified that can exert biological functions. For instance, Chen's work [10] revealed that gastric cancer growth and metastasis can be suppressed by targeting of WWP1 by miR-129-5p and-3p. AS one of the first-characterized hypertrophy-related miRNAs, miR-195 is evidently upregulated in both failing human hearts and hypertrophic mouse hearts [11]. In addition, circulating miR-195-3p (a mature form of miR-195) could be a biomarker of heart failure [12]. Furthermore, overexpression of Pre-miR-195 stimulates hypertrophy, which consequently results in chronic heart dysfunction [11]. Silencing miR-195 can reduce diabetes-induced cardiac hypertrophy and coronary vascular dysfunction [13]. Thus, considerable evidence suggests that miR-195 is a key regulator of hypertrophic process and represents a potential therapeutic target. However, miR-195-3p and miR-195-5p individual role remains unclear. Hence, comprehensively examining mature miR-195 pairs function in the cardiac hypertrophy may provide instructive information about regulatory networks in relation to this process.

This research experiment objective is to explore miR-195-3p/5p role in cardiac hypertrophy and propose a possible mechanism. An* in vivo* model was created by infusing mice with Ang II, and another* in vitro* model was constructed through Ang treatment to H9c2 cells. In addition, the cellular and molecular effects of miR-195-5p/3p on cardiomyocytes, as well as potential downstream targets of miR-195-5p, were explored.

## 2. Materials & Methods

### 2.1. Constructing an Animal Model

C57BL/6 mice (male, 6-8 weeks old and 20±2 g) were maintained under a specific pathogen-free condition with free access to tap water and regular mice chow pellet. A cardiac hypertrophic mouse model was established using an Ang II infusion as described previously [14]. Briefly, 50 mg/kg sodium pentobarbital was used to anesthetize the mice, followed with implantation of 1.46 mg/kg/d of Ang II with osmotic minipump (Alzet model 2002; DURECT, Cupertino, CA) for 14 days in the scapular area. Saline infused mice served as sham group. Blood pressure (systolic and diastolic), were taken using tail-cuff method that is noninvasive (CODA System, Kent Scientific, Torrington, CT) at baseline and 14 days after the Ang II infusion. After 2 weeks, cardiac dimensions and function were analyzed by echocardiography as described below. Then, the mice were sacrificed by cervical dislocation. The hearts were immediately excised, washed in phosphate-buffered saline, gently dried, and weighed. Afterwards, ventricles were separated and stored for histological analysis and RNA/protein isolation.

### 2.2. Echocardiography

Using pentobarbital (50 mg/kg intraperitoneal) the mice were anesthetized, and using dedicated small-animal high-resolution ultrasound system (Prospect, S-Sharp, Taipei, Taiwan), and 40-MHz probe evaluation of both cardiac dimensions and functions was done. To assess LV anatomical and functional parameters the M-mode measurements were taken at the left ventricle papillary muscle short-axis view. We measured the wall size, cardiac dimensions (end-systolic and end-diastolic) together with the heart ejection fraction (EF), and ventricular fractional shortening (FS) as previously described [15]. All reported results were averaged over measurements of three consecutive cardiac cycles.

### 2.3. Histology Analysis

4% paraformaldehyde was used to fix the excised heart tissues and then dehydrated in alcohol. Afterwards, on suctioning into 7 *μ*m slices according to routine procedures the tissues were paraffin embedded. Standard hematoxylin and staining by eosin (H&E) were used to process the tissue and then visualized under light microscopy.

### 2.4. Cell Culture, Cell Treatment

H9c2 cells (a rodent cardiomyocyte cell lines) were procured at the American Type Culture Collection (ATCC) and then routinely kept in a DMEM medium containing 100 IU/mL of penicillin–streptomycin and 10% fetal bovine serum (FBS). The incubation was at a temperature of 37°C, 95% air, and 5% CO2 humid environment. The cardiomyocytes were treated in various concentrations of Ang II (0.1-10 *μ*M) (Merck Millipore, Billerica, MA, USA) for 48 h in order to determine the optimal concentration to induce cell hypertrophy.

### 2.5. Measurement of Cell Surface Area

Cell size was examined under optical microscopy (Olympus model, Tokyo, Japan). To measure the area of each cell, Image J software was used. At least 50 randomly selected cells were measured for each of three independent experiments. All data collected were averaged for further statistical analysis.

### 2.6. Cell Transfection

GenePharma (Shanghai, China) was used to synthesize the miR-195-5p mimic and its inhibitor, miR-195-3p mimic and its inhibitor, and their corresponding negative controls (NC-mimic/NC-inhibitor). The sequence information for synthetic miRNAs and scramble controls were listed in Table 1. H9c2 cells were then transfected by use of Lipofectamine 2000 reagent (Invitrogen, Carlsbad, CA) with either miRNA mimics, inhibitors, or NCs using the manufacturer's procedure.

### 2.7. Western Blot

Cell lysis buffer for western blot and IP (Beyotime Institute of Biotechnology, China) were used to extract all the proteins. The concentrations of proteins were determined with a BCA protein assay kit from the Beyotime Institute of Biotechnology. SDS-PAGE was used to separate equal quantities of total proteins (30 *μ*g per lane) before transferring onto the polyvinylidene fluoride (PVDF) membrane after which they were blocked for 1 hour with 5% skimmed milk. The PVDF membranes were then incubated at a temperature of 4°C overnight with anti-Mitofusion2, anti-Fbxw 7, and anti-GAPDH antibodies (Abcam, USA). After that, TBST was used to wash the membranes and further incubated at room temperature with secondary antibodies, horseradish peroxidase-conjugated goat anti-rabbit, or goat anti-mouse for period of 2 h. Resulting bands were visualized by ChemiDoc XRS+ system from Bio-Rad, USA.

### 2.8. Quantitative Real-Time PCR (qRT-PCR) Analysis

All the RNA in cardiac tissues or cardiomyocytes were extracted by use of total RNA rapid extraction kit (Generay Biotechnology, Shanghai, China) as per the company protocol. Before qPCR, by use of One-Step PrimeScript miRNA cDNA Synthesis Kit (Takara, Dalian, China) and HiScript II Q RT SuperMix for qPCR (Vazyme, Nanjing, China), the RNA was reverse transcribed to miRNA cDNA and total cDNA, respectively. The expression levels of mRNA and miRNA were quantitatively analyzed using qPCR on a Bio-Rad CFX96 Real-Time System (Bio-Rad, Hercules, CA, USA) and SYBR Green master mix (Vazyme, Nanjing, China) which were normalized against U6 and actin mRNA, respectively. To calculate the fold change of target gene's expression, the 2-ΔΔCT method was used. GenePharma (Shanghai, China) was used to design and synthesize the primers of miR-195-5p and also those of miR-195-3p. Primers applied in RT-PCR experiments are listed in Supplemental file 1: Supplemental Table 1.

### 2.9. Luciferase Reporter Assays

For luciferase assays, 80 nmol/L of each mimics (miR-195-5p or the NC) was cotransfected into H9C2 cells with 0.8 *μ*g of pmirGLO vector containing wild-type or mutant 3′-UTR of MFN2 or FBXW7 using ExFect 2000 Reagent (Vazyme, Nanjing, China). 48 h after transfection, by following the company's protocol for Dual-Luciferase Reporter Assay System of Promega, WI, USA, Luciferase reporter assays were done. Normalization of firefly luciferase activity to renilla luciferase activity was done.

### 2.10. Statistical Data Analysis

From at least three independent experimental replicates and as a mean ± SD, data was collected and analyzed using SPSS version 17.0 software. Unpaired Student's t test was used to perform comparison of parameters between the two sets/group. Using analysis of variance (ANOVA) multiple groups comparisons were realized and obeyed least significant difference (LSD) test. The p-value of 0.05 was set to represent statistical significance level of two-tailed p-value.

## 3. Results

### 3.1. MiR-195-5p Upregulation in AngII-Induced Cardiac Hypertrophy

**Figure 1:**
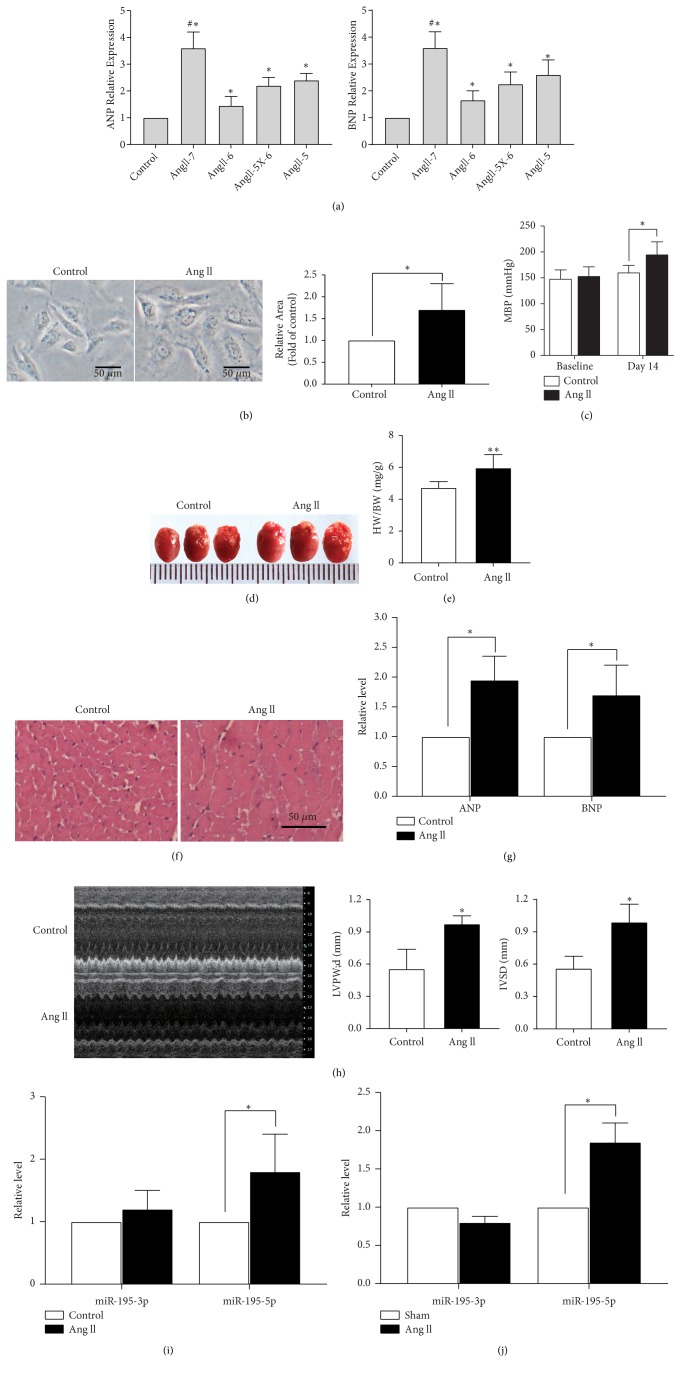
*The hypertrophic myocardium and cardiomyocytes miR-195-3p/-5p expression*. (a) The effects from different concentration of Ang II on ANP, BNP mRNA in H9c2 cells. (b) Representative images of H9c2 cardiomyocytes treated with Ang II or without (control) Ang II (scale bar: 50 *μ*m). (c) Tail-cuff method was used to monitor blood pressure at baseline and 14 days after starting the angiotensin II infusion or sham surgery. MAP: mean arterial pressure. (d) The change of gross morphology of a mouse model heart in hypertrophy induced by AngII-infusion. (e) Comparison of heart-to-body ratio between the Ang II infusion models (n = 5) and sham mice (n = 5). (f) Histological analysis of the heart tissue from different groups using H&E staining (50*μ*m). (g) ANP and BNP mRNA expression in heart tissue were measured by the qRT-PCR assay. (h) The echocardiography results in two-week Ang-II infusion models and the sham group (n = 5). (i) Expressed miR-195-5p together with miR-195-3p were detected by RT-PCR in AngII-induced cardiomyocyte hypertrophy. (j) In hypertrophic heart tissue, miR-195-5p together with miR-195-3p were determined by qRT-PCR. The data is obtained as the mean ± SD; *∗*,* p* < 0.05; ^#^,* p<*0.05 compared to all other groups; n= 5 or 3 different cultures.

First,* in vivo* and* in vitro* models for AngII-induced hypertrophy were created to allow determination of miR-195-3p/5p expression in cardiac hypertrophy. In accordance to Ang II treatment, 5 groups of the H9c2 cardiomyocytes were created in the following order: the control group, the 10^−7 ^mol/l Ang II group, the 10^−6 ^mol/l Ang II group, the 5*∗*10^−6 ^mol/l group, and the 10^−5 ^mol/l Ang II group. Using the first group (control) as a reference, the ANP and BNP mRNAs levels were significantly higher after 10^-7 ^mol/l Ang II treating for in 48 h (Figure 1(a)). Besides, stimulation for 48 h with this concentration induced a substantial increase in the cell size (Figure 1(b)). Thus, 10^-7 ^mol/l was used as the standard condition in the following experiments. In C57BL/6 mice using Ang II (1.46 mg/kg/d, 14d) hypertrophy animal model was achieved. After two weeks of chronic infusion of Ang II, gross morphology, the histological changes, hypertrophic biomarkers, and echocardiography parameters of the mice were examined. The mean blood pressure (MBP) after Ang II infusion for 14 days is remarkably higher than that of the sham group (Figure 1(c)). The AngII-treated group showed substantial increase in the HW/BW (heart-to-body-weight ratio) in comparison to the sham-infused (Figures 1(d) and 1(e)). Furthermore, there was significant expansion in AngII-treated hearts ventricular tissues cross-sectional area (Figure 1(f)), and ANP and BNP levels of expression were upregulated (Figure 1(g)). Also, echocardiography study revealed that hypertrophic indicators including interventricular septum depth (IVSd) and, the diastolic left ventricular posterior wall depth (LVPWd), were noticeably elevated in the AngII-treated mice (Figure 1(h)).

In order to measure the miR-195-3p and miR-195-5p expression in cardiac hypertrophic models, qRT-PCR was performed. The miR-195-5p levels were considerably upregulated in hypertrophy of cellular and mouse models that was AngII-induced (Figures 1(i) and 1(j)). There was upregulation of miR-195-3p expression* in vivo *condition and downregulation* in vitro*, but the change did not reach the significance threshold (Figures 1(i) and 1(j)).

### 3.2. Regulation of Cardiac Hypertrophy by miR-195-5p* In Vitro*

**Figure 2:**
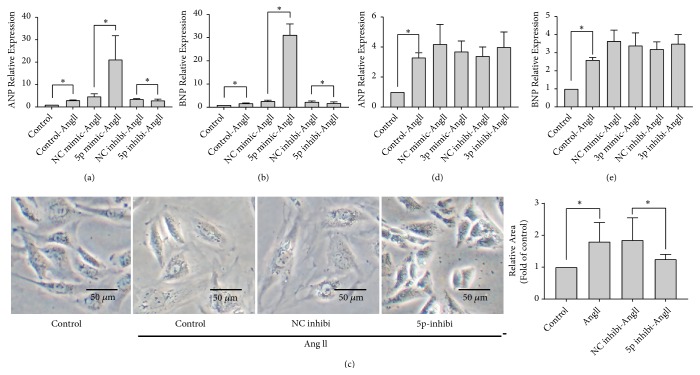
*The miR-195-3p/-5p effect on hypertrophy induced by AngII in H9c2 cells*. (a, b) In AngII-stimulated H9c2 cardiomyocytes after both miR-195-5p mimics and inhibitors transfecting, the ANP and BNP mRNA expression levels were measured using qRT-PCR. (c) Changes of cell surface area in AngII-treated cells with or without the inhibitor of miR-195-5p. (d, e) Effect of miR-195-3p mimic/inhibitor on expression status of ANP and BNP in induced-hypertrophic cardiomyocytes. The presentation of data is as the mean ± SD; *∗*,* p* < 0.05, and n = 3.

To examine the role played in cardiac hypertrophy by miR-195-3p/-5p, we transfected H9c2 cardiomyocytes with miRNA mimics or inhibitors of miR-195-3p/-5p prior to Ang II stimulation. The overexpressed miR-195-5p led to more significant AngII-induced cardiomyocytes hypertrophy, which was assessed by hypertrophic genes' (ANP, BNP) mRNA levels (Figures 2(a) and 2(b)). To further confirm whether miR-195-5p could be a regulator of cardiac hypertrophy, we performed knockdown experiments. Upon induction of cardiac hypertrophy by Ang II, miR-195-5p inhibitors effectively suppressed the ANP and BNP mRNA expression levels, decreasing relative cell areas (Figures 2(a)–2(c)). We also explored whether miR-195-3p had some cardiac hypertrophy regulator roles* in vitro*. In presence of Ang II, overexpression of miR-195p-3p induced a modest albeit not statistically significant reduction in the expression of hypertrophic genes (Figures 2(d) and 2(e)). In contrast, knockdown of miR-195-3p can slightly increase the expression levels of hypertrophy-related genes mentioned above, in comparison to the group of NC inhibitor under Ang II stimulation (Figures 2(d) and 2(e)). As the results suggest that miR-195-5p is capable of promoting cardiac hypertrophy in the AngII-induced cell model, we conducted further research about molecular mechanism underlying its biological role.

### 3.3. Screening of MiR-195-5p Target Genes

**Figure 3:**
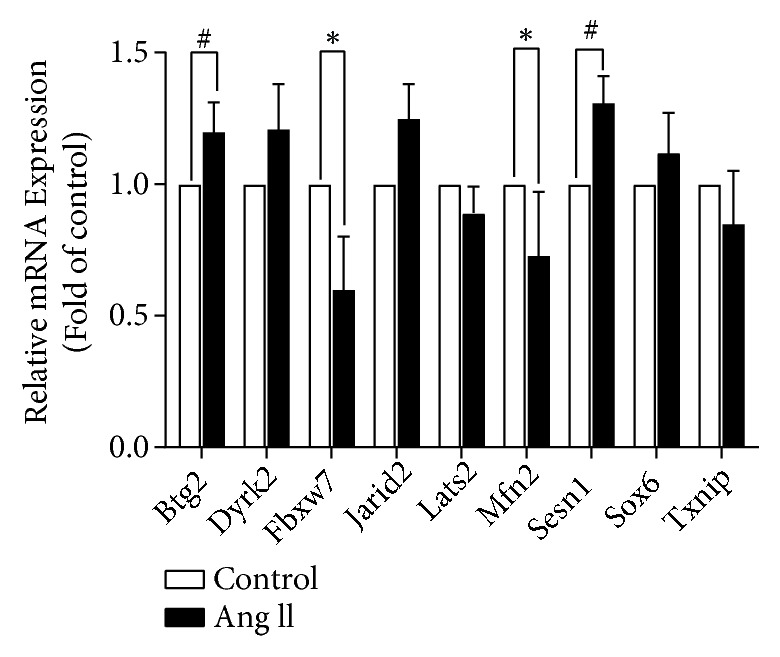
*The potential miR-195-5p target genes expression levels in H9c2 cells at Ang II or no Ang II treatment*. *∗* indicates significant decline vs. control which is* p *< 0.05; # indicates significant increase vs. control which is* p* < 0.05; n = 3.

Because miRNAs exert their functions mainly through inhibiting target genes, a bioinformatics approach (http://www.targetscan.org) had to be employed in deciphering the information of multiple genes that can bind with miR-195-5p. Nine genes with high degrees of integration were selected for further screening: BTG2, SESN1, DYRK2, JARID2, SOX6, FBXW7, TXNIP, LATS2, and MFN2. To further screen target genes, we first investigated the expression levels of the predicted genes in AngII-stimulated H9c2 cardiomyocytes. With the upregulated expression of miR-195-5p, it is expected that its predicted gene targets would be downregulated in the AngII-induced cellular model of hypertrophy. Among these 9 gene candidates, qPCR analyses indicated that FBXW7 and MFN2 were significantly downregulated in the AngII-induced cell model (Figure 3), so these two genes were identified as potential target genes that required further confirmation.

### 3.4. Inhibition of Expression of FBXW7 and MFN2 by MiR-195-5p on Binding to Their 3′UTRs

**Figure 4:**
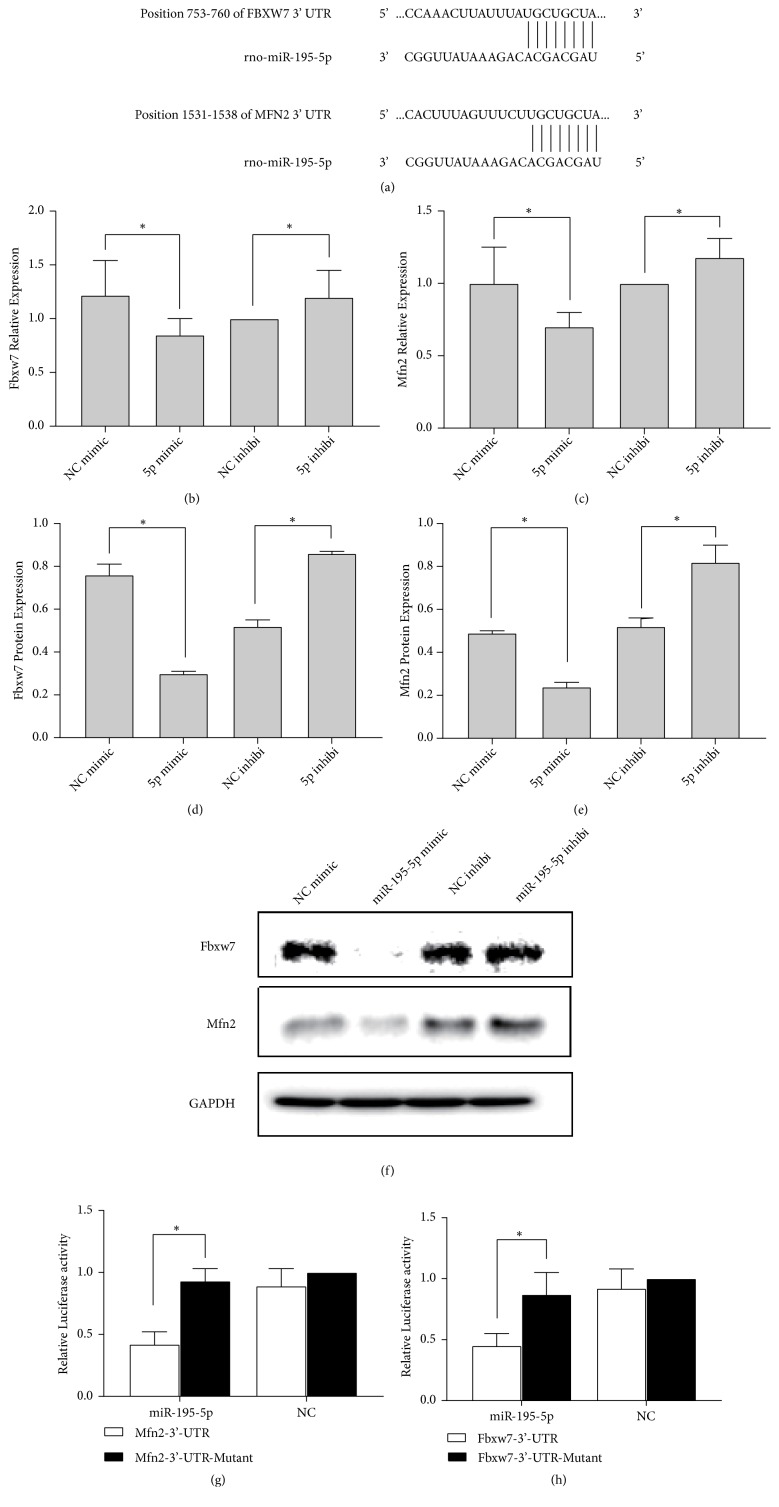
*Targeting of miR-195-5p to the 3*′*-UTR of MFN2 and FBXW7*. (a) The rat miR-195-5p sequence alignment with the MFN2 and FBXW7 3′-UTR region. (b, c) The qRT-PCR analyses of miR-195-5p mimic/inhibitor transfected MFN2 and FBXW7. (d-f) The MFN2 and FBXW7 levels of protein expression in H9C2 cells under different conditions. (g, h) The H9c2 cardiomyocytes were transfected with reporter plasmid and mimic of miR-195-5p/NC-mimic for 2 days. Relative luciferase intensity was determined with a luciferase reporter assay. The data is shown as the mean ± SD; *∗*,* p* < 0.05, n = 3.

To further investigate how miR-195-5p relate to potential target genes (Figure 4(a)), we studied using qRT-PCR and Western blot analysis, respectively, how miR-195-5p affects the expression of FBXW7, and MFN2 levels in H9C2 cardiomyocytes, and it was established that when miR-195-5p was overexpressed, it significantly inhibited mRNA (Figures 4(b) and 4(c)) and protein expression (Figures 4(d)–4(f)) level of FBXW7 and MFN2, whereas its inhibitor had opposite effects (Figures 4(b)–4(f)).

Furthermore, to demonstrate how possible miR-195-5p can directly suppress MFN2 expression, plasmids containing either MFN2 3′-UTR mutant or WT (MUT-pGL3-MFN2 and WT-pGL3-MFN2, respectively) of luciferase reporter were constructed. The results demonstrated miR-195-5p mimic repressed MFN2 3′-UTR reporter gene luciferase activity, with the reporter gene linked to the 3′-UTR of mutant MFN2 abolishing this inhibitory effects (Figure 4(g)). The luciferase reporter assay for FBXW7 also showed similar results as for MFN2 (Figure 4(h)). Taken together, miR-195-5p could directly target and inhibit MFN2 or FBXW7.

## 4. Discussion

Among the major independent predictors of cardiovascular disease (CVD) survival is cardiac hypertrophy [16]. Understanding its mechanism is crucial for developing improved strategies for disease prevention and treatment. In the past decade, numerous data has demonstrated critical role performed by miRNAs in regulating hypertrophy or heart failure. For example, it has been reported that overexpression of miR-208a increases cell size and hypertrophic genes' expression; on the other hand, knockdown of miR-208a can inhibit cardiac hypertrophy [17]. Ganesan's group [18] found that miR-378 displays a protective role against cardiomyocyte hypertrophy by suppressing the MAPK signaling pathway on several distinct levels. In our research, studies on principles of miR-195-5p/-3p mechanisms in cardiac hypertrophy at the functional and molecular level were performed.

Olson's lab [11] first reported the part miRNAs plays in the cardiac hypertrophy, notably, showing miR-195 alone as sufficient enough to promote pathological cardiac hypertrophy upon pre-miR-195 been overexpressed in transgenic mice. Two mature miRNAs (miR-195-3p and miR-195-5p) can be processed from miR-195 precursor by Dicer [19]. With different seed sequences these two miRNAs target different mRNAs and may exert distinct or cooperative functions [20–22]. Despite many studies on miRNA in hypertrophy, the comparative roles of two mature miRNAs originated from the same precursor (such as miR-195-5p and miR-195-3p) have not yet been fully evaluated. Zheng et al. [13] showed that knocked-down miR-195-5p expression attenuated hypertrophy in STZ-induced diabetic mice. In this research, both* in vitro* and* in vivo* models for cardiac hypertrophy induced by Ang II showed miR-195-5p expression considerably upregulated. Overexpression for miR-195-5p significantly promoted AngII-induced cardiac hypertrophy. Meanwhile, the H9c2 cellular miR-195-5p inhibition attenuated AngII-induced hypertrophic responses. Cardiac hypertrophy is possibly regulated by miR-195-5p as indicated by results. As for a part played by miR-195-3p, by analyzing expression of miRNAs, we have revealed that miR-195-3p levels were slightly dysregulated in both cardiac hypertrophy for mouse model and H9c2 cells induced by Ang II. Additionally, there was a trend toward decreased hypertrophic marker genes expressed due to the Ang II presence with miR-195-3p mimic pretreatment.

Wang's group [23] found that cardiac hypertrophy* in vivo* induced by TAC could be aggravated by miR-195a-3p, which may appear to contradict our findings* in vitro*. Notably, Wang's lab [23] showed that for the cardiomyocytes under both normal and hypertrophic conditions the levels of miR-195a-5p expressed were significantly higher than levels of miR-195a-3p. Wang's results also showed that miR-195a-3p is part of impairment in* in vitro* endothelial cell angiogenesis. The overexpression of miR-195-3p could decrease myocardial capillary density and subsequently aggravate coronary blood flow* in vivo*. Therefore, a possible explanation for inconsistent findings could be that overexpression of miR-195a-3p* in vivo* could integrate both a prohypertrophic role on endothelial cells and a possible antihypertrophic role on cardiomyocytes but eventually lead to net promoted cardiac hypertrophy. Further investigations are needed for confirmatory evidence.

Then we further investigated the downstream molecular mechanism through which miR-195-5p regulates the process of hypertrophy. It has been revealed that miRNAs modulate the expression of target genes in different pathways [24, 25]. Through the luciferase reporter assays and the bioinformatics analysis, it was revealed MFN2 and FBXW7 were specifically miR-195-5p targeted. Furthermore, the endogenic mRNA and expressed protein intensities for MFN2 and FBXW7 were significantly altered by miR-195-5p mimic and inhibitor, suggesting that MFN2 and FBXW7 are direct downstream targets of miR-195-5p.

MFN2 (Mitofusin 2), an outer mitochondrial membrane GTPase, has a prominent role on determining structure and homeostasis of the mitochondria [26]. Recent studies [27, 28] have shown a key negative regulator role by MFN2 in cardiac hypertrophy via inhibiting mitochondrial membrane depolarization and ROS (reactive oxygen species) production. Our future work will focus on whether miR-195-5p regulates the integrity and function of the mitochondria by targeting MFN2 in cardiac hypertrophy. In human cancer, F-box and WD-40 domain protein 7 (FBXW7) is member of F-box protein family that acts as classical tumor suppressor [29]. A previous study [30] reported that FBXW7 negatively regulates physical cardiac hypertrophy by inhibiting prohypertrophic HIFIF-1*α*-Postn signaling pathways and eventually promoting inactivation of Akt. In addition, overexpression of miR-195-5p was shown to promote angiogenesis by targeting FBXW7/NOTCH1 signaling [31], and inhibition of NOTCH1 signaling can abolish the antihypertrophic role of Olmesartan [32], indicating that the miR-195-5p-FBXW7-NOTCH1 pathway may be involved in the hypertrophic process. Therefore, a more detailed research is needed to reveal the underlying mechanisms of the role played by miR-195-5p in cardiac hypertrophy process.

## 5. Conclusion

Conclusively, this study shows miR-195-5p as a prohypertrophic miRNA, upregulated in AngII-induced cardiac hypertrophy. Moreover, there is negative regulation of expression of FBXW7 and MFN2 by miR-195-5p through directly targeting the 3′UTRs of these two genes. Future investigations are needed to establish a more comprehensive and in-depth understanding of the role played in hypertrophy process by miR-195-3p, as well as a more detailed cardiac hypertrophy regulation by miR-195-5p underlying mechanisms.

## Figures and Tables

**Table 1:** Sequence information for synthetic miRNAs.

miRNA(GenePharma)	Sequence(5′-3′)
rno-miR-195-3p mimic	sense: CCAAUAUUGGCUGUGCUGCUCCA
	anti-sense: GAGCAGCACAGCCAAUAUUGGUU
rno-miR-195-3p inhibitor	UGGAGCAGCACAGCCAAUAUUGG
rno-miR-195-5p mimic	sense: UAGCAGCACAGAAAUAUUGGC
	anti-sense: CAAUAUUUCUGUGCUGCUAUU
rno-miR-195-5p inhibitor	GCCAAUAUUUCUGUGCUGCUA
mimics negative control	sense: UUCUCCGAACGUGUCACGUTT
	anti-sense: ACGUGACACGUUCGGAGAATT
inhibitor negative control	CAGUACUUUUGUGUAGUACAA

## Data Availability

The data used to support the findings of this study are available from the corresponding author upon request.

## Supplementary Materials

Supplementary Materials Supplemental Table 1. Real-time PCR primer sequences.
