# Supplementary material for: MiR-195-5p Promotes Cardiomyocyte Hypertrophy by Targeting MFN2 and FBXW7
Source: Biomed Res Int. 2019 Jun 25;2019:1580982. doi: 10.1155/2019/1580982 (PMC6614993; doi:10.1155/2019/1580982)
Supplement: Supplementary Materials — Supplemental Table 1. Real-time PCR primer sequences. [file 1580982.f1.pdf]

Supplemental Table 1. Real-time PCR primer sequences

Gene	Forward Primer(5'→3')	Reverde Primer(5'→3')
Mouse-ANP	TCCTCGTCTTGGCCTTTTG	CTCATCTTCTACCGGCATCTTC
Mouse-BNP	GAGACCTCAAAATTCCAAGG	TCTTTCTGCCTTGTGAAGG
Mouse-ACTIN	CTGTCCCTGTATGCCTCTG	ATGTCACGCACGATTTCC
Mouse-U6	GCTTCGGCAGCACATATACTAA	AACGCTTCACGAATTTGCGT
Rat-ANP	CTCCGATAGATCTGCCCTCTTG	GGTACCGGAAGCTGTTGCAGC
Rat-BNP	TGATTCTGCTCCTGCTTTTC	GTGGATTGTTCTGGAGACTG
Rat-ACTIN	CCCATCTATGAGGGTTACGC	TTTAATGTCACGCACGATTTC
Rat-U6	GCTTCGGCAGCACATATACTAAAAT	CGCTTCACGAATTTGCGTGTCAT
Rat-Btg2	TCAAGGTTTTCAGTAGGGCG	TGTGGTTGATGCGGATACAG
Rat-Sox6	CCTCACCACATAAGCCTGAC	TTCAGCGTATCCACCACATC
Rat-Sesn1	GGACGGGCTTTCAAATGGTG	CCGCTTCTCTCTCTCACGTC
Rat-Dyrk2	AGTTCGCCCACTCCATTCTG	CTCCGACCCTGCTGCTTTAA
Rat-Fbxw7	TATTGAGACCGGCCAGTGTT	GCTCCACTAACAACCCTCCT
Rat-Jarid2	ACTGCCAGTACCTGCTTTCC	TGGTGGTCGTTCTCTGTGTG
Rat-Lats2	GGACCCCAGGAATGAGCAAA	TGGTGGGATGGGAATGCTTC
Rat-Mfn2	CTCTGTGCTGGTTGACGAGT	AGCGGTCAGACATGTTTCGT
Mmu-miR-195-3p	GGCCAATATTGGCTGTGC	GAGAGGAGAGGAAGAGGGAA
Mmu-miR-195-5p	AGGGTAGCAGCACAGAAAT	TTGGGAGGTAGGAGGTTGAT
Rno-miR-195-3p	CGTTATCCTAGCAGCACAGAAAT	TATGGTTTTGACGACTGTGTGAT
Rno-miR-195-5p	TCTCGCCCAATATTGGCTG	TATGGTTGTTCACGACTCCTTCAC
